# HC diet inhibited testosterone synthesis by activating endoplasmic reticulum stress in testicular Leydig cells

**DOI:** 10.1111/jcmm.14143

**Published:** 2019-03-18

**Authors:** Chunxiao Yu, Fangjie Jiang, Meijie Zhang, Dandan Luo, Shanshan Shao, Jiajun Zhao, Ling Gao, Changting Zuo, Qingbo Guan

**Affiliations:** ^1^ Department of Endocrinology Shandong Provincial Hospital Affiliated to Shandong University Shandong Provincial Key Laboratory of Endocrinology and Lipid Metabolism, Institute of Endocrinology and Metabolism Shandong Academy of Clinical Medicine Jinan Shandong P. R. China; ^2^ Department of Rehabilitation Xinhua Hospital Affiliated to Shanghai Jiaotong University School of Medicine Shanghai P. R. China; ^3^ Scientific Center Shandong Provincial Hospital affiliated to Shandong University Jinan Shandong P. R. China; ^4^ Department of Gynaecology and Obstetrics Shandong Provincial Hospital Affiliated to Shandong University Jinan Shandong P. R. China

**Keywords:** endoplasmic reticulum stress, HC diet, hypercholesterolaemia, Leydig cell, testosterone deficiency

## Abstract

Emerging epidemiological studies indicate that hypercholesterolaemia is a risk factor for testosterone deficiency. However, the underlying mechanism is unclear. Testicular Leydig cells are the primary source of testosterone in males. To identify the effect and mechanism of cholesterol overload on Leydig cell function, rats were fed with a HC (HC) diet to induce hypercholesterolaemia. During the 16‐week feeding period, serum testosterone levels were reduced in a time‐dependent manner in rats fed the HC diet. Accordingly, these steroidogenic enzymes within the Leydig cells, including steroidogenic acute regulatory protein (StAR), cholesterol side‐chain cleavage cytochrome P450 (P450scc) and 3β‐hydroxysteroid dehydrogenase (3β‐HSD), were down‐regulated. Notably, the HC‐fed rats showed evident endoplasmic reticulum (ER) stress in the testis, including a dilated ER as an evident pathological change in the Leydig cell ultrastructure, up‐regulated ER stress biomarker (binding immunoglobulin protein) levels and activation of the activating transcription factor 6 (ATF6)‐related unfolded protein response pathway. Further analysis showed that when 4‐phenyl butyric acid (4‐PBA) was used to block ER stress in HC‐fed rats for 8 weeks, the testosterone deficiency was significantly alleviated. Our findings suggested that high dietary cholesterol intake affected serum testosterone levels by down‐regulating steroidogenic enzymes and that activated ER stress might serve as the underlying mechanism.

## 1 INTRODUCTION

Testosterone, as an important steroid hormone, plays vital roles in multiple physiologic pathways.^1^ The deficiency of testosterone is not restricted to andrology and endocrinology; it is also considered to be a risk factor for metabolic diseases and disorders, coronary heart disease^2^ and other chronic diseases including cancer.^3^, ^4^ Testosterone deficiency (TD), also known as hypogonadism, is characterized by decreased serum testosterone concentration or activity and is a condition affecting a substantial proportion of men. Ageing is strongly associated with declining testosterone levels. However, there is great interindividual variability in testosterone levels, with the global prevalence of TD varying from 7% to 49% in ageing men.^5^ Modifiable lifestyle factors including a western‐style diet and diseases such as type 2 diabetes are considered to be strongly associated with declining testosterone levels.^4^, ^6^

Cholesterol is an essential component of the human diet and performs an important role as a raw material in the synthesis of steroid hormones and vitamin D.^7^ A substantial amount of evidence indicates that hypercholesterolaemia is a crucial risk factor for mortality and morbidity, not only for cardiovascular disease^8^, ^9^ but also for non‐alcoholic fatty liver disease,^10^ cancer^11^ and Alzheimer’s disease^12^, ^13^ Our previous epidemiological study demonstrated that low testosterone was related to elevated total cholesterol (TC) in serum from 4114 middle‐aged and older Chinese participants,^14^ which was consistent with previous research.^15^ Several animal studies have also observed that hypercholesterolaemic rats^16^ and mice^17^ showed low serum testosterone levels. These findings imply that hypercholesterolaemia is an important risk factor for testosterone deficiency, but the underlying mechanism is not obvious.

Testicular Leydig cells, which reside in the testis interstitium, are the primary source of testosterone in males. Testosterone synthesis is initiated by cholesterol transport into the mitochondria via StAR, which has been considered the rate‐limiting step. Next, cholesterol is converted to pregnenolone in the mitochondria by P450scc. Pregnenolone then moves from the mitochondria into the smooth endoplasmic reticulum (ER) and is converted to testosterone by several steroidogenic enzymes, including 3β‐HSD, cytochrome P450 17A1 (P450c17) and 17β‐hydroxysteroid dehydrogenase (17β‐HSD). These enzymes play necessary roles in Leydig cell function to maintain steroidogenesis. Several studies reported that high‐fat diets or high‐calorie diets could regulate Leydig cell steroidogenesis by several mechanisms, including dysregulation of the hypothalamic‐pituitary‐gonadal axis, increases in the leptin signal transduction pathway and inflammatory cytokine release in the testis.^18^, ^19^, ^20^ These studies suggested that impaired testosterone synthesis might be an important mechanism in the process of diet affecting testosterone levels. Hence, in this study, we aimed to investigate adverse effects and mechanisms on Leydig cell steroidogenesis in mice fed a HC diet.

The ER is a crucial organelle responsible for steroid biosynthesis and maintenance of cellular homeostasis. Many disturbances that cause accumulation of misfolded proteins and the unfolded protein response (UPR) trigger ER stress.^21^ Binding immunoglobulin protein (BiP) is the best‐characterized ER chaperone that directly interacts with all ER stress sensors and maintains them in inactive forms in non‐stressed cells. Under stress, the unfolded proteins promote the dissociation of glucose‐regulated protein (GRP78)/BiP by inducing the phosphorylation and relocalization of the ER transmembrane sensors, inositol‐requiring protein 1α (IRE1α), activating transcription factor 6 (ATF6) and protein kinase RNA (PKR)‐like ER kinase (PERK), which triggers subsequent signalling pathways to exert their effects.^22^ As is known, the cholesterol content in membranes is tightly controlled. Even minimal increases in cholesterol content in the ER membrane can impair ER protein‐folding capacity and trigger ER stress,^21^ damaging the functions of the ER such as steroid biosynthesis. Some researchers have reported that ER stress participates in testicular dysfunction caused by a high‐calorie diet and type 2 diabetes mellitus.^20^, ^23^ However, whether ER stress is involved in the pathogenesis of cholesterol overload‐induced testosterone deficiency is unclear.

In the present study, to determine the effect of cholesterol overload on TD, the rats were fed with a HC diet to assess histopathologic changes, Leydig cell function and ER stress biomarkers in the testes. In addition, the rats were fed with a HC diet combined with treatment with the ER stress blocker 4‐PBA to reveal the underlying mechanism. This study demonstrates a crucial role of ER stress in the dysfunction of HC diet‐induced Leydig cell testosterone synthesis.

## 2 METHODS

### 2.1 Animals and treatment

All animal experiments were approved by the Animal Ethics Committee of Shandong Provincial Hospital and performed according to the Shandong Provincial Hospital Animal Care and Use Committee. Male Sprague Dawley rats (170‐190 g) were purchased from Vital River Laboratory Animal Technology Co., Ltd. (Beijing, China). After adaptation to the housing conditions for 1 week, the rats were randomly divided into two groups and assigned to one of the following two diets for 16 weeks: (a) normal control diet (n = 30): 100% standard rodent chow (NC group, 14.09 kJ/g; Beijing Keao Xieli Feed. Co., Ltd.) or (b) HC diet (n = 30): 98.7% standard rodent chow supplemented with 1% cholesterol and 0.3% sodium cholate (HC group, 13.54 kJ/g; Beijing Keao Xieli Feed. Co., Ltd.). The composition of the experimental diets was assayed by the Beijing Research Institute for Nutritional Resources, and details are shown in Table 1.

**Table 1:** Composition of experimental diets

Ingredients	Normal control diet	HC diet
Protein, g/100 g	20.1	19.7
Water, g/100 g	9.2	9.3
Fat, g/100 g	6	6.7
Selenium, g/100 g	1.4 × 10^−5^	1.6 × 10^−5^
Cholesterol, g/100 g	0.02	1.096
Fatty acids, g/100 g	5.28	5.25
Total kJ/g	14.09	13.54

For further mechanistic analysis, 40 rats were randomly assigned into four groups and fed with a normal or HC diet. Simultaneously, the ER stress inhibitor 4‐PBA (100 mg/kg/day) (Sigma‐Aldrich, MO, USA) or an equal volume of physiological saline (PBS) was administered to the rats by intraperitoneal injection for 8 weeks. The design of the animal models is illustrated in Figure S1.

All mice were adapted to a 12‐hour light/dark cycle at 22‐25°C, and bodyweights were monitored weekly during the feeding period. Fasting blood samples were obtained by subclavian venous puncture, and the serum was isolated between 8:00 and 10:00 am every 4 weeks. Two separate batches of animals were killed after 16 or 8 weeks of treatment. The testes were quickly excised and properly collected. One testis was fixed in 3% glutaraldehyde and 1% osmium or 4% paraformaldehyde tetroxide for morphological analysis and immunohistochemistry; the other testis was stored in liquid nitrogen for assessment of mRNA, protein expression and cholesterol content.

### 2.2 Lipid parameter and sex hormone analysis

Serum levels of triglycerides and TC were measured using enzymatic methods with Olympus reagents and automated spectrophotometry performed on the Olympus AU5400 system (Olympus Corporation, Tokyo, Japan).

For the detection of testosterone, testicular tissues (10 mg) were homogenized by sonication in phosphate‐buffered solution (PBS, 100 μL) and centrifuged. The supernatant was collected for intratesticular testosterone concentration assessment. Serum total testosterone, intratesticular testosterone (Uscn Life Science & Technology Co., Ltd., Wuhan, China) and serum LH (LSBio, Seattle, USA) levels were measured using an enzyme‐linked immunosorbent assay (ELISA). Testosterone concentrations in testicular tissues were normalized to protein concentrations, which were determined using a BCA protein assay kit (Shenneng Bocai Biotechnology Co., Ltd., Shanghai, China). All procedures were carried out in accordance with the instructions provided by the manufacturers.

### 2.3 Filipin staining

The sections of the testis (5 μm), frozen in OCT embedding medium, were stained with filipin fluorescence dye according to the manufacturer’s instructions (GenMed Scientifics Inc, USA). Images were captured using a microimaging system (Axio Imager A2; Zeiss, Jena, Germany).

### 2.4 Free cholesterol content assay

Free cholesterol was extracted from the testis and assayed according to the manufacturer’s instructions (Applygen Technologies, Inc, Beijing, China). All data were normalized to the concentration of proteins from the same sample.

### 2.5 Haematoxylin and eosin staining and light microscopic analysis of testis histology

The tissues were fixed for 24 hours in 4% paraformaldehyde and then dehydrated through an ethanol series, cleared in xylene and embedded in paraffin. The paraffin blocks were consecutively sliced into 5‐mm thick sections, at least 4‐5 sections per sample. Some of the slides were stained with haematoxylin and eosin (H&E) according to the manufacturer’s instructions. Images were captured using a microimaging system to observe the histopathological changes in the testis tissue, at least five consecutive fields for each section.

### 2.6 Transmission electron microscopy

The testes were fixed in buffered 3% glutaraldehyde and 1% osmium tetroxide, dehydrated through an ethanol series and embedded with epoxy resin. Ninety nanometre sections were cut consecutively using an LKB‐V ultramicrotome (LKB, Bromma, Sweden) and double stained with uranyl acetate and lead citrate. The ultrastructure of the testicular gland was observed and photographed by a specialist who was blinded to the study using a transmission electron microscope (H‐800; Hitachi, Tokyo, Japan).

### 2.7 Immunohistochemistry and immunofluorescence

Testis sections were prepared as described for H&E staining, followed by heat treatment in Tris‐EDTA buffer for antigen retrieval. For the immunohistochemistry assay, all procedures were carried out following standard techniques using a goat anti‐3β‐HSD primary antibody (1:100). For the negative control, IgG was added instead of the primary antibody. The reactivity of the antibodies was detected using a streptavidin‐peroxidase histostain‐SP kit (Zhongshan Golden Bridge Biotechnology Co., Ltd.). The peroxidase activity was visualized with diaminobenzidine, followed by haematoxylin staining to counterstain the nuclei. Further, the area of Leydig cells relative to the local area was analysed using ImageJ. For the immunofluorescence assay, the slides were blocked with 5% donkey serum albumin prior to the addition of the primary antibody, rabbit anti‐StAR (1:100), and then incubated overnight at 4°C. After that, slides were incubated with FITC‐conjugated goat anti‐rabbit IgG (1:2000; Thermo Fisher) for 1 hour at room temperature. The nuclei were stained with DAPI (1:1000; Invitrogen). After treatment, at least five consecutive fields of each slice were observed under the microimaging system. Antibodies are shown in Table S1.

### 2.8 RNA isolation and Real‐time PCR

RNA was extracted from the testis tissue by using TRIzol reagent (Takara, Tokyo, Japan). The RT reaction system (20 μL) consisted of 1 μg of total RNA, oligo‐dT primer, random 6 mers and reverse transcriptase (RT) (Takara). PCR experiments were performed with an LC480 (Roche, Mannheim, Germany). SYBR Green (DBI) was used to detect the amplification of cDNA with absolute quantitation. Each reaction system was composed of 10 μL of SYBR green, 1 μL of cDNA sample, 1 μL of each primer pair (5 pmol/μL) and 8 μL of distilled water. Thermal cycling conditions were 30 seconds at 95°C followed by 45 cycles at 95°C for 5 seconds, 60°C for 20 seconds and 72°C for 25 seconds. The PCR primers used to amplify the steroidogenic enzymes are listed in Table 2. β‐actin was employed as an endogenous control to normalize the data. The specificity of the amplified products was verified by melting curves. Each sample was analysed in triplicate, and samples from different groups (n = 6‐8) were analysed to determine the value.

**Table 2:** Primers sequences used for the analysis of mRNA expression levels in rats

Gene	Gene bank no.	Product (bp)	Forward primer	Reverse primer
StAR	NM_031558.3	142	CCTGAGCAAAGCGGTGT	TGATGATGGTCTTTGGCAGC
Cyp11a1	NM_017286.2	140	TTACACAGACGCATCAAGCAGCAA	GGGTCCACGATCTCCTCCAACAT
Hsd3b	NM_001007719.3	131	AACTGCCACTTGGTCACACTGTC	GTCCCGATCCACTCCGAGGTTT
Cyp17a1	NM_012753.2	127	GATGGATGCACAGGCTGAGGTTAG	TAGGAGGAAGGAGGACCGTAGGAG
Hsd17b3	NM_054007.1	145	TGACCAAGACCGCCGATGAGTT	TGGGTGGTGCTGCTGTAGAAGAT
β‐actin	NM_031144	99	CTAAGGCCAACCGTGAAAAGA	CCAGAGGCATACAGGGACAAC

### 2.9 Protein extraction and immunoblot analysis

Testicular samples were homogenized in RIPA buffer containing protease inhibitors and phosphatase inhibitors (Shenergy Biocolor Bioscience & Technology Company, Shanghai, China), subjected to ultrasound pyrolysis and centrifuged at 15 000 *g* for 15 minutes. The protein content was measured using a BCA assay. After denaturation, protein samples were subjected to SDS‐PAGE, electrotransferred to polyvinylidine fluoride membranes (Millipore, Billerica, MA, USA) and blocked in 5% non‐fat powdered milk in Tris‐buffered saline containing 0.1% Tween 20 (TBST) for 1 hour. The membranes were then incubated with specific antibodies overnight at 4°C, including those against steroidogenic key enzymes, namely, StAR, P450scc, 3β‐HSD and P450c17; ER stress‐related signal pathway proteins, namely, BiP, p‐PERK, t‐PERK, p‐eIF2α, t‐eIF2α, ATF4, p‐IRE1α, t‐IRE1α, sXBP1, CHOP and ATF6; and using β‐actin for normalization. After three washes in TBST, the membranes were incubated with the appropriate secondary antibodies conjugated with horseradish peroxidase (HRP) for 1 hour and visualized by a HyGLO HRP detection kit (Denville, NJ, USA). Protein expression levels were quantified with Fluor Chem Q SA software. Antibodies are shown in Table S1.

### 2.10 Statistical analysis

All data were analysed with SPSS 19.0 and are presented as the mean ± SD. Means were compared using unpaired Student’s *t* test for comparisons between two groups and one‐way ANOVA for comparisons among multiple groups. A two‐tailed value of *P* < 0.05 was considered statistically significant.

## 3 RESULTS

### 3.1 Effect of HC diet on serum lipid profiles and testosterone levels in the rats

**Figure 1:**
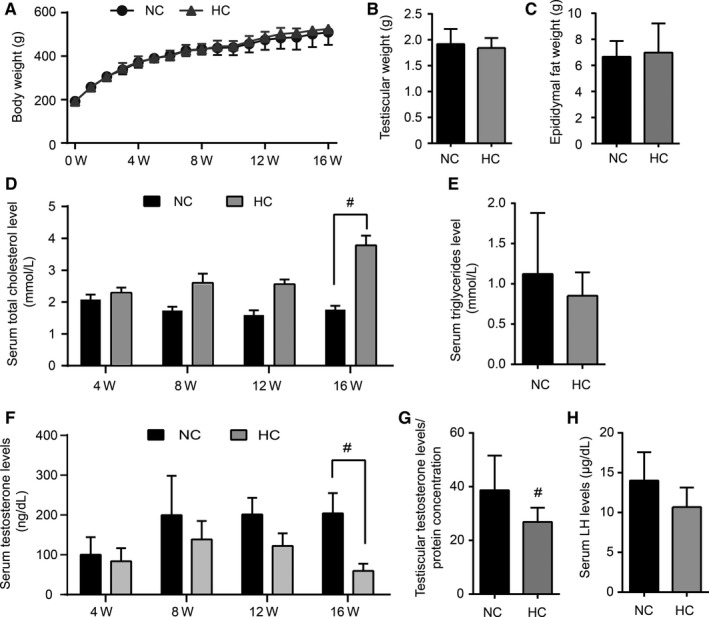
The high‐cholesterol (HC) diet increases serum cholesterol level and decreases testosterone level in rats. Rats were treated with a normal control diet (NC group) or a HC diet (HC group) for 16 wk. During the feeding period, the bodyweight of these rats was monitored weekly (A), and the serum total cholesterol (D) and testosterone (F) levels of the rats were assayed every 4 wk. In addition, testicular weight (B), epididymal fat weight (C), serum triglyceride (E), intratesticular testosterone (G) and LH (H) levels were detected at the 16th wk. Data points are presented as the mean ±SEM (n = 8 per group). #*P* < 0.05 vs the NC group

During the 16‐week feeding period, there was no significant difference in bodyweight gain, testicular weight and epididymal fat weight between the rats in the HC group and NC group (Figure 1A‐C). To observe the effects of the HC diet on circulating lipid profiles, serum levels of TC and triglycerides were examined. As shown in Figure 1D, as the duration of the feeding time of the HC diet was prolonged, serum cholesterol levels continued to increase. At the 16th week, there was an approximately twofold increase in serum cholesterol levels in the HC group compared with those in the NC group (1.76 ± 0.35 vs 3.78 ± 0.91, *P* < 0.001). No significant difference was seen in serum triglycerides levels among the rats in both groups (Figure 1E).

To assess the effect of the HC diet on testosterone synthesis in rats, serum testosterone and LH were detected dynamically during feeding time. At the 4th, 8th and 12th weeks, the serum testosterone levels showed a decreasing trend in the rats treated with the cholesterol diet. After feeding for another 4 weeks (16th week), serum testosterone levels were observed to dramatically reduce by approximately 70% compared with those of the NC group (204.5 ± 50.53 vs 59.73 ± 18.05, *P* = 0.04, Figure 1F). The intratesticular testosterone levels were further analysed at the 16th week. Similar to the testosterone level in serum, the intra‐testis testosterone levels in the HC group decreased significantly compared with those in the NC group (*P* = 0.04, Figure 1G). However, there was no significant difference in the level of serum LH at the starting and ending time points (Figure 1H).

### 3.2 The HC diet‐induced abnormal cholesterol accumulation and ultrastructure in the testicular Leydig cells

**Figure 2:**
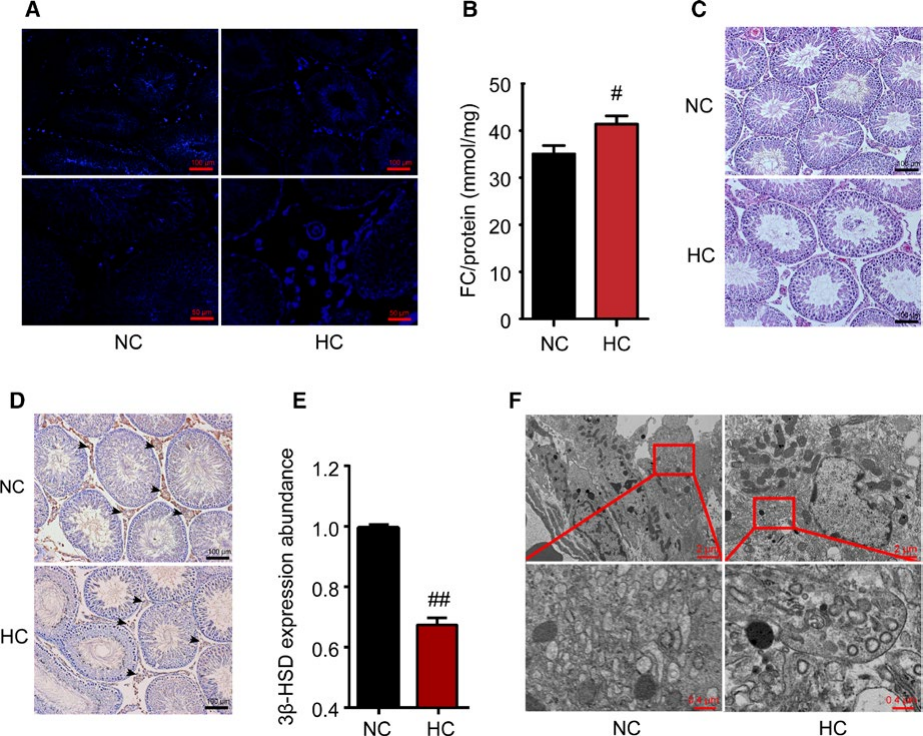
HC diet induces morphological changes in the testes. After HC‐diet feeding, filipin fluorescence was performed to display free cholesterol deposition in the testis gland in both groups (blue colour) (A). Further, testicular free cholesterol (FC) content was assayed and corrected by the total protein content (B). Representative photomicrographs of H&E staining of testicular sections are shown (C). D, Testis was immunostained for 3β‐HSD which is specifically expressed in Leydig cells, and 3β‐HSD expression abundance in the Leydig cells was analysed (E). The ultrastructure of the Leydig cells in the testis of the NC group revealed a normal fine structure. In the testes of the HC‐group rats, the dilated endoplasmic reticulum (red box) was observed in the Leydig cells (F). Data are presented as the mean ±SEM (n = 8 per group). #*P* < 0.05, ##*P* < 0.01 vs the NC group. All results were from at least three independent experiments

To investigate the effect of HC diet on cholesterol content in the testis gland, filipin fluorescent staining, which can accurately reflect the accumulation and location of free cholesterol, was performed to visualize lipid content in Leydig cells. Strong staining was exhibited in the interstitial Leydig cells of the HC‐group rats (Figure 2A). Consistent with the above results, the free cholesterol content of the testis in the rats in the HC group was significantly greater than that of the rats in the NC group (*P* = 0.019, Figure 2B).

Testicular histomorphological changes were evaluated by H&E staining. As shown in Figure 2C, compared with the NC group, the number and diameter of seminiferous tubules were not affected by the HC diet; however, the arrangement of the tubules was loosened and displayed a lower thickness of the germinal epithelium in HC‐diet mice. The testicular Leydig cells were further assessed by 3β‐HSD immunohistochemical staining, which is a marker of Leydig cells. The expression abundance of 3β‐HSD in testicular Leydig cells in the HC group was also significantly reduced in comparison with those in the NC group (Figure 2D,E).

Testicular Leydig cells contain abundant ER, mitochondria and lipid droplets to support testosterone production. A substantial number of smooth ER arrange as anastomotic tubular networks throughout the cytoplasm. Electron micrographs showed that one notable feature within the cytoplasm of the Leydig cells from HC‐group rats was swollen mitochondria with widened cristae. Furthermore, the smooth ER was dilated and drastically decreased in the Leydig cells compared with those in the NC group (Figure 2F). These results indicate that the accumulation of cholesterol disrupted the general morphology of the testis and might influence the function of the Leydig cells.

### 3.3 The HC diet decreased steroidogenic enzyme expression in the testis

**Figure 3:**
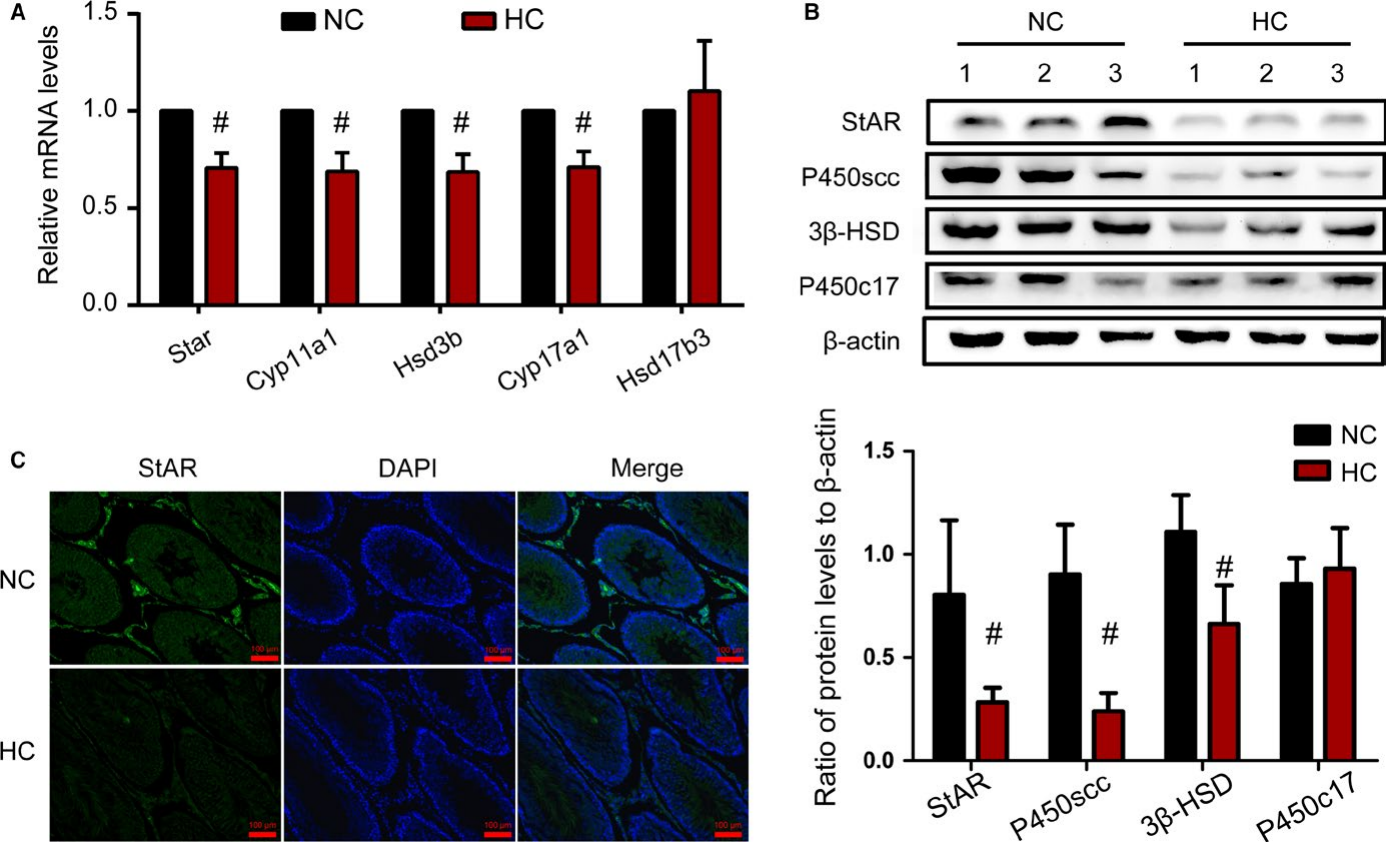
Expression of steroidogenic enzymes decreased by the HC diet in Leydig cells. By quantification with real time PCR analysis and normalization to β‐actin levels in the testis, relative mRNA levels of Star, Cyp11a1, Hsd3b, Cyp17a1 and Hsd17b3 are shown compared with those in the NC group (A). Protein expression of StAR, P450scc, 3β‐HSD and P450c17 was measured by western blotting, with relative protein abundance analysis (B). Representative images of immunofluorescence staining performed in the testicular Leydig cell for StAR, which is a key enzyme in steroidogenesis (C). Data were expressed as the mean ±SEM (n = 8 per group). #*P* < 0.05 vs the NC group. All results were from at least three independent experiments

Testosterone biosynthesis is a complex process in which the key enzymes StAR, P450scc, 3β‐HSD, P450c17 and 17β‐HSD are involved. To examine the influence of a HC diet on the enzymes related to the testosterone synthesis pathway in rat Leydig cells, we detected the expression of these molecules by RT‐PCR, western blotting and immunofluorescence. The corresponding genes of these proteins are Star, Cyp11a1, Hsd3b, Cyp17a1 and Hsd17b3. First, as shown in Figure 3A, the expression of steroidogenic genes, except for Hsd17b3, was reduced at the RNA level in rat testis in the HC group compared with that in the NC group (*P* < 0.05). Moreover, it is more striking that the protein expression of StAR, P450scc and 3β‐HSD was significantly decreased in the testis of HC‐diet rats compared with that in the testis of NC rats (Figure 3B). Furthermore, as we had expected, the immunofluorescence staining results showed that the StAR protein was distributed in the interstitium of the testis and that the fluorescence intensity of StAR was clearly decreased in the HC group compared with the NC group (Figure 3C). Taken together, these results indicate that the HC diet decreased steroidogenic enzyme expression, which might lead to a decrease in the function of testosterone synthesis in rat.

### 3.4 The HC diet enhanced expression of ER stress biomarkers in the testis

**Figure 4:**
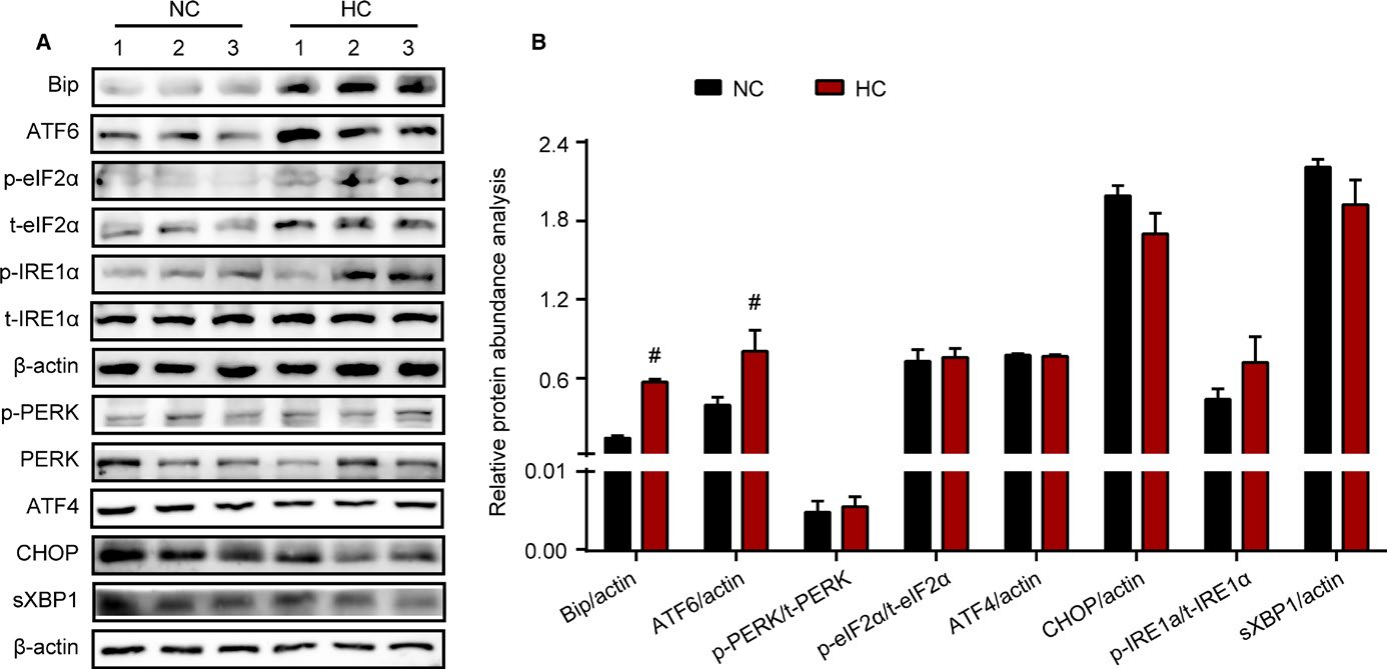
Enhanced ER stress participated in the process of HC diet‐induced testicular dysfunction. Representative protein expression for BiP, p‐PERK, total‐PERK (t‐PERK), p‐eIF2α (at Ser51), total‐eIF2α (t‐eIF2α), p‐IRE1α (at Ser724), total‐IRE1α (t‐IRE1α), sXBP1, CHOP, ATF4 and ATF6 was measured by western blotting in both treated groups (A). Relative protein density between p‐PERK and t‐PERK, p‐eIF2α and t‐eIF2, p‐IRE1α and t‐IRE1α, BiP, sXBP1, CHOP, ATF4, ATF6 and β‐actin was analysed (B). Data were expressed as mean ±SEM (n = 8 per group). #*P* < 0.05, ##*P* < 0.01 vs the NC group. All results were from at least three independent experiments [Colour figure can be viewed at wileyonlinelibrary.com]

ER stress plays an important role in the response to cell stress. Based on the ER injury in Leydig cells observed by electron microscopy, ER stress biomarker expression and phosphorylation were evaluated by western blotting to analyse whether the HC diet‐induced ER stress in rat testis. As shown in Figure 4A,B, the expression of BiP and ATF6 was significantly increased in the testis of the HC group compared with that in the testis of the NC group, while the phosphorylation and protein expression of IRE1α‐ and PERK‐related pathways, including the phosphorylation of IRE1α, eIF2α and PERK, sXBP1, CHOP and ATF4, were not significantly increased. These results indicated that ER stress and the ATF6‐related UPR pathway were induced in the testis of HC‐diet rats. This change might be one of the factors causing dysfunctional steroidogenesis in Leydig cells.

### 3.5 Blocking ER stress alleviated HC diet‐induced Leydig cell dysfunction

To further illustrate the role of testicular ER stress and explore possible treatment strategies in HC diet‐induced Leydig cell dysfunction, 4‐PBA, which is a chemical chaperone inhibiting ER stress, was administered to the rats by intraperitoneal injection. The testosterone levels in serum and testosterone biosynthesis function in testis were monitored.

**Figure 5:**
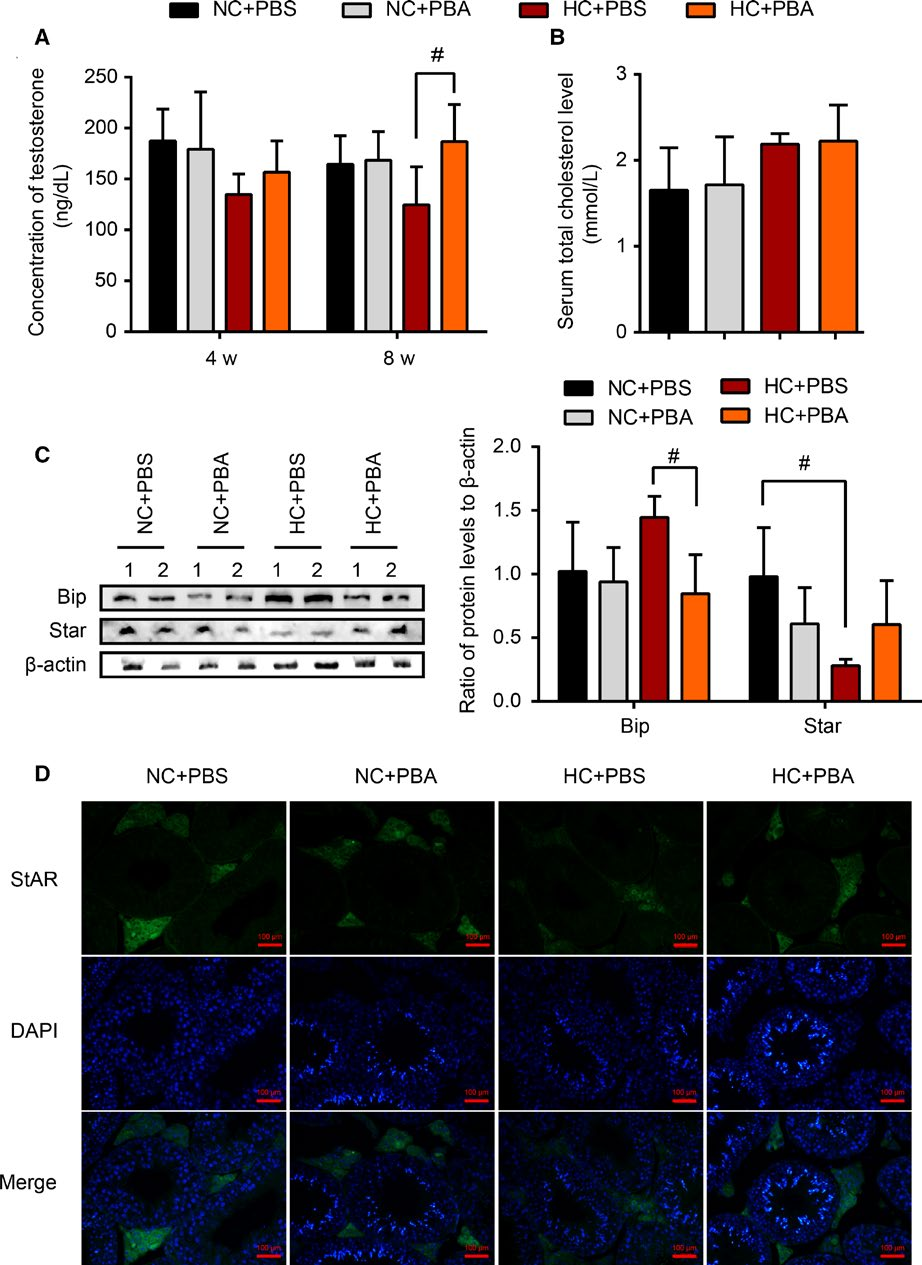
The improvement of Leydig cell function when blocking the ER stress. HC diet‐fed rats were treated with 4‐PBA and PBS for 8 wk. The levels of serum testosterone (A) were assayed after 4 and 8 wk of treatment. At the same time, the serum total cholesterol levels at the 8th wk (B) were determined. Representative protein expression and protein density analysis of the ratios among BiP, StAR and β‐actin were shown in the treated groups at the 8th wk (C). Representative images of immunofluorescence staining performed in the testes for StAR are shown for all treated groups (D). Data were expressed as the mean ±SEM (n = 10 per group). #*P* < 0.05 vs the HC group. All results were from at least three independent experiments.

As shown in Figure 5A, serum testosterone levels decreased beginning in the 4th week in the rats fed with a HC diet, but in rats administered with both a HC diet and 4‐PBA, serum testosterone levels were observed to increase and were similar to normal levels at the 8th week (*P* < 0.05, compared with the HC group). Further, the ER stress response and steroid synthesis function in the testis were tested. As expected, the expression of BiP in the testis was significantly reduced after administration of 4‐PBA compared to that in the HC group (Figure 5C,D). At the same time, following treatment with 4‐PBA, the inhibitory effects on the expression of StAR in Leydig cells induced by a HC diet were reversed (Figure 5C,D). These results illustrated that ER stress was involved in the HC diet‐induced Leydig cell dysfunction, which can be alleviated by blocking ER stress.

## 4 DISCUSSION

It is well known that testosterone is essential for multiple physiological pathways including male secondary sexual development and fertility.^24^ The Leydig cells, which are located between the seminiferous tubules of the testis, are responsible for the synthesis and secretion of testosterone.^25^, ^26^, ^27^ Several studies have reported that dietary factors such as high‐fat diets and high‐calorie diets could inhibit Leydig cell steroidogenesis.^18^, ^20^ Cholesterol is an essential component of the human diet. At normal levels, cholesterol performs an important role as a raw material in the synthesis of steroid hormones.^7^ A growing body of evidence has indicated that overload of cholesterol is a crucial risk factor for mortality and morbidity.^8^, ^9^, ^10^ However, the effects and mechanisms of cholesterol overload on Leydig cell steroidogenesis lack thorough investigation. In the present study, we demonstrated that a HC diet could induce cholesterol accumulation in testicular Leydig cells. Subsequently, ER stress and the ATF6‐related UPR pathway were induced, accompanied by a decrease in steroidogenic enzyme expression, resulting in reduced testosterone production. Importantly, blocking ER stress with 4‐PBA could alleviate the dysfunction in HC diet‐induced Leydig cell steroidogenesis.

To investigate the effects of cholesterol overload on testicular Leydig cells, a standard HC diet, excluding high fat and high calories, was fed to rats in the present study. A significant increase in serum levels of TC, but not TG, was found in rats fed the HC diet. This finding indicated that the HC diet‐induced hypercholesterolaemia in rats, which is consistent with a report by Yang et al^28^ A HC diet has been reported to dramatically increase cholesteryl ester lipid droplets in segments 1 and 2 of the caput epididymal epithelium in lxr^−/−^ mice, leading to less viable and motile spermatozoa.^29^ Similarly, in our study, increased cholesterol deposition was found in testicular tissues corresponding with the increased serum TC levels of rats fed with a HC diet. Free cholesterol deposition was more obvious in Leydig cells, probably because of the HC diet and the ability of Leydig cells to utilize the cholesterol. These results suggested that a HC diet promotes cholesterol accumulation in testicular Leydig cells.

It is known that testosterone release is pulsatile in nature.^30^ Hence, we collected blood during the same time frame to avoid the influence of fluctuating hormone levels. The levels of serum testosterone were detected. As expected, the serum testosterone levels showed a decreasing trend beginning in the 4th week and significantly progressively decreased in rats after HC diet feeding to the 16th week. Correspondingly, the level of testosterone in the testis also decreased significantly. These results demonstrated that HC diets might have long‐term and chronic toxic effects on the synthesis of testosterone. In this research, ELISA based the antibody‐based immunoassays was used to detect the testosterone concentration, it could not be excluded that testosterone levels were over‐estimated due to limited specificity towards androgen metabolites by antibody‐based method. Intriguingly, it is well known that obesity and type 2 diabetes are risk factors for male testosterone deficiency.^23^, ^31^ In the rat model fed with a HC diet, in addition to elevated serum cholesterol levels, bodyweight and blood glucose levels (data not shown) did not rise compared with those in the normal control diet rats. These results indicated that HC diet‐induced male testosterone deficiency might be due to cholesterol overload, rather than the combination of hyperglycaemia and obesity.

Testicular Leydig cells are the primary source of testosterone in males.^26^ Testosterone is synthesized from cholesterol, which is mediated by pituitary gonadotropin LH through multiple signalling pathways, including StAR, P450scc, 3β‐HSD, P450c17 and 17β‐HSD in Leydig cells.^32^ Several studies have mentioned that dysregulation of the hypothalamic‐pituitary‐gonadal (HPG) axis played a role in the reduction in the release of testosterone.^33^, ^34^ In our research, to evaluate the effect of the HPG axis on the HC diet‐induced reduction in testosterone level, the pituitary hormone LH was detected. As the results showed, the HC‐fed rats manifested lower serum testosterone levels not accompanied by reduced serum LH levels. These findings demonstrate that the dysregulation of the HPG is not considered a reason for the HC diet‐induced male testosterone deficiency observed in this study. The function and number of testicular Leydig cells are closely related to testosterone production.^25^ Some studies have reported that diet and environmental factors could lead to apoptosis and regulate steroidogenesis in Leydig cells, decreasing testosterone production.^18^, ^20^, ^35^ To further analyse the mechanism of the HC diet‐induced reduction in testosterone levels, testicular histomorphological changes and 3β‐HSD, which are localized only in the Leydig cells and often used as a Leydig cell marker,^36^ were analysed. The weaker staining of 3β‐HSD of the Leydig cells suggested that the function of the steroidogenic cells was disturbed by cholesterol overload. The expression of steroidogenic enzymes at the transcriptional and translational levels in the rat testis was further evaluated. Accompanied by reduced testosterone levels, testicular tissues from HC diet‐fed rats exhibited lower expression levels of StAR, P450scc and 3β‐HSD compared to those in control samples. These results indicate that Leydig cell dysfunction is involved in HC diet‐induced male testosterone deficiency.

Although cholesterol is an essential substrate for testosterone biosynthesis, excess cholesterol can be toxic.^37^ Free cholesterol accumulation can increase the free cholesterol/phospholipid ratio in cellular membranes and form needle‐shaped cholesterol crystals, leading to a consequent dysfunction of integral membrane proteins and cellular organelle disruption.^21^ The mitochondria and ER are crucial organelles responsible for steroid biosynthesis in Leydig cells.^38^ The ultrastructure of the testicular gland was further observed by using a transmission electron microscope in this research. Consequent to testicular free cholesterol deposition, HC diet‐fed rats exhibited evident dilated ER in the Leydig cells, which means that ER dysfunction might be present. The ER is a central cellular organelle in which transmembrane and secretory proteins are synthesized, folded, post‐translationally modified and transported. Changes in ER lipid composition under cholesterol overload may disrupt ER membrane functions and cause an accumulation of misfolded and unfolded ER proteins, which can trigger ER stress. Growing evidence supports the proposal that HC diet could cause ER stress. For example, cholesterol loading triggered the activation of the UPR in hepatic L02 cells,^39^ and the activation of ER stress has been reported to play a role in HC diet‐induced cognitive deficits^13^ and aortic valve calcification^40^ in mice. These findings indicate that the changes in ER dilation may be associated with enhanced ER stress in testicular Leydig cells induced by HC feeding.

Binding immunoglobulin protein is the best‐characterized ER chaperone that directly interacts with all ER stress sensors and maintains them in inactive forms in non‐stressed cells. In our study, the important molecular chaperone BiP was significantly up‐regulated in testicular Leydig cells of HC‐treated rats, further supporting the idea that ER stress is involved in Leydig cell dysfunction. Once three transmembrane ER sensors are activated by BiP under a stress situation, the UPR response can reduce the protein load entering the ER, lower protein synthesis and induce a transcriptional program to increase the capacity of the ER and resolve the stress. Actually, the UPR stress sensors may have distinct sensitivities to specific inducers of ER stress.^41^ The up‐regulated expression of sensor ATF6 was evident in the testis of HC diet‐fed rats, indicating the important role of the ATF6 pathway during ER stress. From previous reports and our results, we can conclude that excess cholesterol may act as an inducer of ER stress in the testicular microenvironment.

An increasing number of studies have indicated that ER stress played an important role in the biosynthesis of testosterone by aggravating apoptosis, oxidative stress, etc^20^, ^35^ In this study, the impaired steroidogenic ability of the testicular Leydig cells was accompanied by ER stress in the HC diet‐induced male testosterone‐deficient rats. To further explore the relationship between impaired biosynthesis of testosterone and ER stress in the testicular Leydig cells, a chemical chaperone that stabilizes protein conformation and improves ER protein‐folding capacity, 4‐PBA, was used. To investigate the preventive effect of this ER stress blocker, a HC diet and 4‐PBA were administered to the rats at the same time. The dosage used was the same as that in a study by Guowei Zhang et al, which reported that 4‐PBA injection clearly attenuated dibutyl phthalate‐induced ER stress in the testis.^42^ Serum testosterone levels were dynamically monitored from the 4th week. Intriguingly, after using 4‐PBA as a chaperone for 8 weeks, the reduced testosterone levels induced by a HC diet were obviously relieved compared to those in the vehicle‐treated HC diet‐fed rats. Further studies confirmed that, consistent with testosterone levels, testicular ER stress and expression of testosterone synthesis key proteins were significantly alleviated. These results not only identify that ER stress is mediated, at least in part, in HC‐induced Leydig cell dysfunction in the testes but also suggest a new potential therapeutic target to prevent and treat HC diet‐induced testosterone deficiency.

In conclusion, we demonstrated that a long‐term HC diet could dramatically decrease the level of testosterone and affect the steroid synthesis function of testicular Leydig cells, which can be rescued with 4‐PBA supplementation. These findings illuminate that ER stress may participate in the dysfunction in steroid‐producing cells induced by cholesterol overload. However, further work should be carried out to unveil the underlying molecular mechanisms by which ER stress influences the steroidogenic ability of the Leydig cells. This research provides a reasonable mechanism and suggests a new potential therapeutic target to prevent and treat HC diet‐induced testosterone deficiency.

## CONFLICT OF INTEREST

Author disclosures: CX Yu, FJ Jiang, MJ Zhang, DD Luo, SS Shao, JJ Zhao, L Gao, CT Zuo and QB Guan declare no conflicts of interest.

## AUTHORS’ CONTRIBUTION

Qingbo Guan and Changting Zuo conceived of the project and designed the research. Chunxiao Yu conducted the research, analysed the data and wrote the main part of the paper. Fangjie Jiang conducted the research and was involved with the writing of the manuscript. Meijie Zhang and Dandan Luo participated in parts of the experiments. Shanshan Shao contributed to feeding the rats. Jiajun Zhao and Ling Gao provided expert input and technical guidance. Qingbo Guan had the primary responsibility for the final content. All authors read and approved the final manuscript.
