# Supplementary figures and images for: HC diet inhibited testosterone synthesis by activating endoplasmic reticulum stress in testicular Leydig cells
Source: J Cell Mol Med. 2019 Mar 18;23(5):3140–50. doi: 10.1111/jcmm.14143 (PMC6484377; doi:10.1111/jcmm.14143)

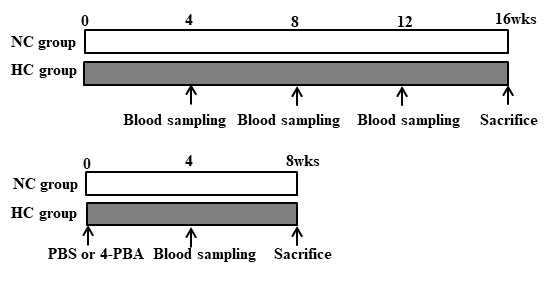

Supplement: Supplementary file 1 [file JCMM-23-3140-s001.tif]
