# Supplementary material for: HC diet inhibited testosterone synthesis by activating endoplasmic reticulum stress in testicular Leydig cells
Source: J Cell Mol Med. 2019 Mar 18;23(5):3140–50. doi: 10.1111/jcmm.14143 (PMC6484377; doi:10.1111/jcmm.14143)
Supplement: Supplementary file 2 [file JCMM-23-3140-s002.docx]

Supplemental Table 1. Antibodies used in Immunohistochemistry, immunofluorescence and immunoblot analysis.

| Antibody | Species | Dilution in immunoblot analysis | Corporation | Catalogue Number |
| --- | --- | --- | --- | --- |
| StAR | rabbit | 1:1000 | Cell Signaling Technology | 8449s |
| P450scc | rabbit | 1:200 | Biosynthesis Biotechnology | bs-3608R |
| P450c17 | rabbit | 1:100 | Biosynthesis Biotechnology | bs-3853R |
| 3β-HSD | goat | 1:100 | Santa Cruz | SC-30820 |
| Bip | rabbit | 1:1000 | Proteintech | 11587-1-AP |
| p-PERK | rabbit | 1:1000 | Cell Signaling Technology | 12185s |
| t-PERK | rabbit | 1:1000 | Cell Signaling Technology | 12185s |
| p-eIF2α | rabbit | 1:800 | Cell Signaling Technology | 9721 |
| t-eIF2α | rabbit | 1:1000 | Cell Signaling Technology | 9722 |
| p-IRE1α | rabbit | 1:1000 | Abcam | ab124945 |
| t-IRE1α | rabbit | 1:1000 | Abcam | ab37073 |
| sXBP1 | rabbit | 1:1000 | Cell Signaling Technology | 83418 |
| CHOP | mouse | 1:1000 | Cell Signaling Technology | 2895t |
| ATF4 | rabbit | 1:1000 | Cell Signaling Technology | 11815s |
| ATF6 | rabbit | 1:1000 | Abcam | Ab37149 |
| β-actin | mouse | 1:7500 | Proteintech | 60008-1 |
